# Mechanical Performance of Biocomposites Based on Straw Fiber Self-Reinforced Plasticized Flours of Bread Wheat Grown with Different Nitrogen Fertilization Management Strategies

**DOI:** 10.3390/polym17101347

**Published:** 2025-05-15

**Authors:** Paolo Benincasa, Franco Dominici, Francesca Luzi, Catia Governatori, Mariano Pauselli, Giacomo Tosti, Fabrizio Sarasini, Debora Puglia

**Affiliations:** 1 Department of Agricultural, Food and Environmental Sciences, University of Perugia, Borgo XX Giugno, 74, 06123 Perugia, Italy; mariano.pauselli@unipg.it (M.P.); giacomo.tosti@unipg.it (G.T.); 2 Department of Civil and Environmental Engineering, University of Perugia, Strada di Pentima 4, 05100 Terni, Italy; franco.dominici@unipg.it; 3 Department of Science and Engineering of Matter, Environment and Urban Planning (SIMAU), Polytechnic University of Marche, Via Brecce Bianche 12, 60131 Ancona, Italy; f.luzi@staff.univpm.it; 4 AMAP, Agenzia per l’Innovazione nel Settore Agroalimentare e della Pesca (Marche Agricoltura Pesca), Via Giulio Latini, 64, 60035 Jesi, Italy; governatori_catia@amap.marche.it; 5 Department of Chemical Engineering Materials Environment, Sapienza Università di Roma and UdR INSTM, Via Eudossiana 18, 00184 Rome, Italy; fabrizio.sarasini@uniroma1.it

**Keywords:** *Triticum*, nitrogen, fertilization, thermoplastic flour, composite, tensile properties, crystallinity, humidity

## Abstract

Previous research has demonstrated the possibility to produce wheat flour-based thermoplastics, whose tensile properties depend on flour characteristics that are affected by wheat variety and crop nitrogen (N) fertilization management. This work further investigates the reinforcing effect on thermoplastic composites determined by wheat straw obtained from two wheat varieties (Bologna, BL; Bora, BR) grown under four N fertilization treatments differing in rate and application timing as follows: (1) always well N fed (N300: fertilized with 300 kg N ha^−1^ and split into five applications of 60 kg N ha^−1^ each across the growing cycle), (2) N fed only very early (N60+0: fertilized only in one early application of 60 kg N ha^−1^), (3) N fed only very late (0+120: fertilized only in one application of 120 kg N ha^−1^ at pollination) and (4) never N fed (N0). The finely cut straw was added by 15% (*w:w*) to the flour of treatment N300 of each corresponding wheat variety to produce thermoplastic bulk samples. We performed the analysis of straw composition, FESEM imaging of straw stems, X-Ray diffraction analysis of flours and straws, thermal analysis of straw, and tensile tests on bulk samples. The results demonstrate that, for both cultivars, the reinforcing effect of the straw was maximum when the straw came from crops grown with low and early N availability (i.e., N0 and N60+0) and minimum when the straw came from crops grown with high and late N availability (i.e., N300 and N0+120). The greater reinforcing effect of straw from N0 and N60+0 was likely due to greater stem compactness, higher cellulose proportion and higher crystalline fractions. The reinforcing effect decreased for all plasticized composites when they were stabilized for 48 h at higher ambient humidity (53% RH vs. 11% RH) before performing the tensile tests. Overall, our results confirm that plant-based materials engineering needs to carefully consider the variability of source material characteristics as affected by crop growing conditions.

## 1. Introduction

The rapid evolution of global development has led to shortages of raw materials, an increase in energy costs and a progressive exhaustion of petroleum resources. In this context, there is a growing demand to develop and produce eco-sustainable, ecological and environmentally friendly products from renewable sources, which are easily available and abundant [1,2,3]. In this regard, plant-based materials are among the most readily available renewable sources, given the high productivity of primary producers.

Wheat is the most important crop worldwide for cultivated land, with over 200 million hectares and a production volume of over 700 million tons of grain per year [4]. An average harvest index (i.e., the ratio between the grain and total above-ground biomass) of 0.4 on a world scale may be assumed, which implies at least 1 billion tons of straw produced yearly. The primary use of flour should be for human or animal feeding, and the primary use of straw should be its incorporation into the soil to counteract the decrease in organic matter, which is a key component of soil fertility. The straw can be incorporated directly during seedbed preparation for the subsequent crop or after its use as straw litter in animal husbandry, which comes out as an excellent manure to be returned to the soil. However, several alternative uses have been proposed in recent decades for both flour and straw, including biofuels and bio-based materials.

As far as wheat flour is concerned, Leblanc et al. [5] first proposed the idea to use it for direct plasticization as an energy saving and cost-effective alternative to the plasticization of purified starch. Later on, Puglia et al. [6] and Benincasa et al. [7] found that the properties of thermoplastic films derived from bread wheat flours are strongly influenced by the gluten composition and baking properties of the flour, which in turn depend on the cultivar (contraction of “cultivated variety”) and the crop growing conditions, with water and nitrogen availabilities as key factors [8,9,10]. Moreover, the mechanical performance and composting behavior of wheat flour-derived thermoplastics may be affected by the addition of fiber, such as that provided by the bran at different proportions and grinding levels, since it acts as a reinforcement [11,12]. Generally, the addition of a reinforcement increases the strength of the material but reduces its deformability, making the material suitable for different destinations and uses.

Wheat straw might represent another source of fiber for self-rising wheat flour. The combination of plasticizable starch with raw fibers (both bran and straw) or their extraction products (i.e., cellulose nanocrystals) has already been considered in the literature as a valuable option [13,14,15,16,17,18]. Similarly to what was said above for the grain, straw abundance and characteristics are affected by the cultivar, the environment (rainfall, temperature regime and soil properties) and the cultivation practices (crop density, nitrogen fertilization and crop protection, to say a few). For example, in barley, F1 hybrid lines have been found to have different cellulose content and properties as compared to traditional self-pollinated lines [19]. For a specific cultivar in a specific environment, the N fertilization rate and timing are most likely the key factors influencing either vegetative development or the setting and filling of the grain pool (i.e., the final sink of assimilates) [20], resulting in different biomass accumulation and partitioning. In particular, early N application promotes vegetative growth and thus the proportion of straw, while early N deficiency followed by late N fertilization increases the allocation of biomass to the grains [21]. The effect of the N fertilization schedule also depends on the rainfall regime. In fact, in most temperate countries, wheat is a rainfed crop grown between the fall season and the late spring season, and its growth is subjected to the interaction effect between the N and water availabilities in each season [22]. The different biomass partitioning is not the only effect of the timing of N application, since the structure and composition of straw tissues are also likely to be affected [23,24,25,26].

In a very recent study, Benincasa et al. [27] investigated the effect of N availability on wheat grain development, imposing different N fertilization schedules and varying total N rates and application timings. The grains harvested from each treatment were partly destined to produce thermoplastics, whose mechanical properties have been studied in the work by Benincasa et al. [8]. The straw was also harvested from each treatment with the aim of using it as reinforcement in the plasticization process. The aim of this work was to evaluate the mechanical properties of plasticized wheat flours self-reinforced with the straw fiber of crops grown with different N fertilization rates and timings that were never considered before. In particular, this work aims to investigate the effect of wheat nitrogen fertilization management on the reinforcing effect that crop straw provides to wheat-flour-based thermoplastic composites. The main goal was to give evidence that plant-based materials engineering needs to carefully consider the variability of source material characteristics as they are affected by crop growing conditions. To date, plant-based materials engineering has been considering mainly (or only) the plant species while neglecting the variability arising from the cultivated genotype and the crop growing conditions (soil, climate and agricultural practices).

## 2. Materials and Methods

### 2.1. Origin of the Wheat Straw: The Field Trial and Crop N Fertilization Treatments

The straw of common wheat (*Triticum aestivum* L.) was harvested in June 2018 from a field experiment carried out at the experimental station of the Department of Agricultural, Food and Environmental Sciences of the University of Perugia located in Papiano (43 °N, 12.3 °E, 165 m a.s.l.) in the middle Tiber Valley, Central Italy. Details on the field trial and crop management are reported in Benincasa et al. [27].

Two common wheat cultivars, Bologna (BL) and Bora (BR), were subjected to six different fertilization schedules according to a split-plot design with four replicates (randomized blocks), with the cultivar allotted to the main plot and the N fertilization treatment allotted to the sub-plot. The straw used here for the production of biocomposites was collected from the following four fertilization treatments: (1) always well N fed (N300), i.e., fertilized with 300 kg N ha^−1^ and split into five applications of 60 kg N ha^−1^ each on 16 December (one month after plantlet emergence), 10 January, 12 February (tillering phase), 15 March (before shoot elongation) and 8 April (active shoot elongation); (2) N fed only very early (N60+0), i.e., fertilized only in one application of 60 kg N ha^−1^ on 16 December; (3) N fed only extremely late (0+120 May), i.e., fertilized only in one application of 120 kg N ha^−1^ on 2 May (anther pollination in the main spike); and (4) never N fed (N0).

The grain size and flour technological characteristics for the two cultivars are described in detail in previous publications [8,21,27]. In brief, it is worth noticing that both cultivars are high in protein and have similar values of Chopin’s alveograph parameters, with a high value of W (i.e., the width of the alveogram, which expresses the deformation energy of the dough). Concerning the growth habit, both cultivars have similar plant heights (around 0.85 m) and similar tillering aptitudes (around 1.3) in crops with appropriate seeding density and good water and N availability.

The rainfall during the fall–spring season was very high (over 800 mm from 1 August 2017 to 30 June 2018, of which almost 500 mm was between mid-November and mid-May), and this caused the desired differentiation in crop N availability in the different phases of the growing cycle. In particular, in the two treatments, N60+0 and N0+120, it was possible to obtain the opposite imbalances in N nutrition necessary for the aim of this study. Therefore, differences among N treatments were very marked for both cultivars in terms of crop growth indices, leaf greenness, total above-ground biomass accumulation and partitioning between the straw and grains, as well as in terms of grain yield and quality [8,27]. For these reasons, this experimental year was ideal for the aim of this work, i.e., to evaluate the mechanical performance of thermoplastic composites obtained with straw coming from actually different N availabilities.

### 2.2. Origin of the Wheat Flour Used for Plasticization

The wheat (*Triticum aestivum* L.) flour of the cultivars Bologna and Bora used in this experiment was obtained from the grains harvested in the same field experiment from the N300 treatment. This was, among our experimental treatments, the only treatment that never limited N availability, which is the most common situation in advanced wheat production, although a total rate of 300 kg N ha^−1^ can be considered exceeding the actual crop N requirement. The flour of this N treatment had given good performance of derived thermoplastic films in the work by Benincasa et al. [8], where both the standardized grain milling procedure and the flours characteristics (Chopin’s alveograph parameters, protein content and gluten fraction composition) of treatments for both Bologna and Bora are reported.

### 2.3. Crop Vegetative Growth, Straw Sampling, Composition and Fiber Structure

The growth of the vegetative apparatus, which is supposed to affect straw characteristics, was determined just before harvest by sampling all plants in a 1 m^2^ area per plot and by determining plant height, the number of tillers, and the dry biomass of the straw. The straw was collected by hand from the plant samples used to determine wheat above-ground biomass accumulation and partitioning. The straw was clean enough, and there was no presence of soil particles visible by eye. It is worth noticing that, even when harvested mechanically, the straw is generally quite clean, considering that, in the Mediterranean environment and the harvest period, there is little rain (and thus little risk of mud splats) and provided the stem is cut not too close to the soil surface.

The determination of the dry matter (DM) content of the straw used in this experiment was obtained by mixing samples from all replicates of each treatment, homogenizing bulk samples and drying them in an oven at 105 °C for 24 h. The neutral detergent fiber (NDF, sum of cellulose, hemicellulose and lignin) and the acid detergent fiber (ADF, sum of cellulose and lignin) of the bulk sample of each treatment were determined using the filter bag equipment by Ankom (Ankom Technology 119 Corp., Fairport, NY, USA), following the method described by Van Soest [28]. The methodology comprises the treatment of the sample with a neutral detergent solution and washing with a thermostable amylase to solubilize sugars, starches and pectin. The remaining material consists of hemicellulose, cellulose and lignin. Hemicellulose was solubilized using an acidic detergent solvent. The remaining cellulose and lignin were then treated with concentrated sulfuric acid, dissolving the cellulose (Cell) and separating the lignin (ADL) in the residue. Hemicellulose content was obtained by subtracting ADF from NDF, while the amount of cellulose was measured by subtracting the value of ADL from ADF. Crude protein (CP) was measured following the procedures described in AOAC Official Method 2001.11 [29]

The morphology of wheat stems, which represent the most relevant fiber source in straw, was investigated using a field emission scanning electron microscope (FESEM, Supra 25-Zeiss, Oberkochen, Germany) with a 2.5 kV accelerating voltage. The stems were cut transversely at room temperature and fixed on a conductive sticky tape exposing the cross section, gold sputtered and then analyzed.

### 2.4. X-Ray Diffraction Analysis of Flours and Straws

X-ray diffraction (XRD) analysis was performed with a diffractometer X’Pert Pro by Philips (Malvern Panalytical Ltd., Malvern, UK) (CuKα_ radiation = 1.54060 Å) at room temperature. XRD patterns were collected in the 2θ range between 10° and 80° with a step size of 0.02° and a time per step of 3 s. The obtained diffractograms were analyzed, and the Crystallinity Index was calculated.

### 2.5. Thermal Analysis of Wheat Straw

Thermal degradation of the different wheat straws was carried out via thermogravimetric analysis (TGA, Seiko Exstar 6300, Tokyo, Japan) by adopting a temperature ramp from 30 °C to 600 °C at 10 °C min^−1^. Dynamic thermal tests were carried using samples of about 5 mg under nitrogen flow (200 mL/min). Mass loss (TG) and derivative mass loss (DTG) curves were determined for each tested material.

### 2.6. Plasticization, Injection Molding and Measurements on Bulk Samples

The plasticization of the flour and straw composite samples was performed with an extruder Xplore 5 and 15 Micro Compounder (DSM, Sittard, The Netherlands) using adequate quantities of glycerol, water and other additives to facilitate the process, as per the recipe reported by Benincasa et al. [8]. Stems of the straw obtained from each of the four N fertilization treatments (N0, N60+0, N0+120 and N300) of the cultivars (Bologna, BL, Italy; Bora, BR) were cut to a length of approximately 5 mm using an automatic cutter and added to the flour of treatment N300 of the corresponding cultivar at a ratio of 15% (*w*/*w*). The choice of only one flour per cultivar had the aim of focusing the comparison on the reinforcement effect of straw from different treatments, and the N300 flour was chosen because, as said previously, it had given good performances of derived thermoplastic films in the work by Benincasa et al. [8], and actually, non-limiting crop N availability is the most common situation in advanced wheat production. The mixtures were first mixed at a low speed in a laboratory mixer (planetary mixer at 60 rpm for 3 min). The doughs were then introduced into the extruder and mixed for 6 min at 120 rpm to achieve plasticization. A temperature profile of 135–140–145 °C was selected for the three areas of the extruder, namely feeding, metering and die, since this profile had been proven as the most suitable to obtain good mechanical performance for plasticized wheat flours [6,8].

The refined flours of N300 (i.e., without addition of straw) of both cultivars were also included in the test. An injection molder (DSM Xplore Micro 10cc Injection Moulding Machine, Xplore Instruments BV, Sittard, The Netherlands) coupled to the extruder was employed to produce the specimens. The compounded materials were then forced into a closed mold (set at 150 °C) with a dumbbell shape (1BA type geometry, compliant with the ISO 527-2 standard [30]), followed by an injection step at a pressure of 400 bar for 10 s and a post injection step at 100 bar for an additional 10 s. Therefore, ten different formulations were produced (Table 1), and dumbbell-shaped tensile test specimens were prepared in accordance with ISO 527 standard to investigate the tensile behavior of these biocomposites. The 5 mm length cutting of stems was considered suitable for the experimental conditions needed for the preparation of the short-fiber biocomposites in a lab-scale microextruder. On the other hand, the injection conditions in such a lab-scale equipment do not let the fibers align in thermoplastic flours: the length we considered was suitable to avoid hindering macroscopic processability at the selected filling level, ensuring homogeneous fiber distribution and dispersion within the polymeric matrix.

### 2.7. Tensile Properties of Bulk Samples

The tensile characterization of the thermoplastics obtained from the refined flours (BL_R and BR_R) and from the eight mixtures with straw fibers (BL_S_N0, BL_S_N60+0, BL S_N0+120, BL S_N300; BR_S_N0, BR_S_N60+0, BR_S_N0+120 and BR_S_N300) was carried out in accordance with the ISO 527 standard. Being biomaterials based on plasticized flour, they are extremely sensitive to environmental conditions and to humidity; therefore, tensile tests were carried out on thermoplastic composites stabilized at different air relative humidity levels, as these tests are usually performed in biomaterial engineering. After conditioning the thermoplastic bulk samples in a controlled environment for 48 h at 23 °C with relative humidity levels of 11% (11RH) and 53% (53RH), 5 samples for each formulation and for each of the two relative humidity levels were subjected to tensile tests by using a LR30K Plus universal electronic dynamometer (Lloyd Instruments, Bognor Regis, UK); test were conducted at a crosshead speed of 5 mm min^−1^, and stress–strain characteristic curves (σ–ε) were plotted using the specific software for the dynamometer NEXYGEN Plus Material Testing (Lloyd Instruments, Bognor Regis, UK). Values for Young’s modulus (E), tensile strength (σ_b_) and strain at failure (ε_b_) were calculated.

### 2.8. Statistical Analysis

Data on the mechanical properties of the thermoplastic composites, reported as the mean ± standard deviation, were analyzed using one-way analysis of variance (ANOVA). The OriginPro 9.0 software (Northampton, MA, USA) was used to perform Tukey’s test for paired comparison of means within each cultivar, with a significance level of α = 0.05.

## 3. Results

### 3.1. Crop Vegetative Growth, Straw Stem Morphology and Microstructure

Crop vegetative growth was strongly affected by N management, with similar trends for both cultivars. Just to give an idea, the plant height ranged from 0.58 m for both cultivars in N0 to 0.86 and 0.89 for Bologna and Bora, respectively, in N300. The tillering index ranged between 1 for both cultivars in N0 to 1.3 and 1.4 for Bologna and Bora, respectively, in N300. Straw biomass for Bologna and Bora was 2.5 and 2.1 t ha^−1^, respectively, in N0; 7.2 and 7.0 ha^−1^, respectively, in N60+0; 4.6 and 4.3 ha^−1^, respectively, in N0+120; and 11.6 and 12.0 ha^−1^, respectively, in N300. These differences clearly indicate that N treatments were effective (i.e., the amount and timing of N availability to crops were actually different), further confirming the evidence from the same field experiment for the effect on grain yield and yield components [27] and for the effect on flour baking properties and gluten composition [8].

**Figure 1:**
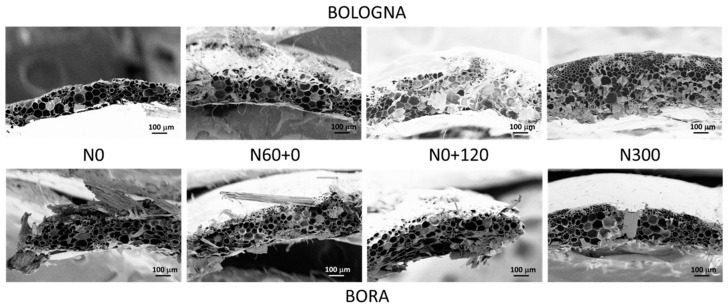
FESEM micrographs (250× magnification) of straw stem sections of the two cultivars of bread wheat (Bologna, above; Bora, below) grown with four different nitrogen (N) fertilization treatments: N0: the unfertilized control, N60+0: N fed only very early with 60 kg N ha^−1^, N0+120: N fed only extremely late (i.e., at pollination) with 120 kg N ha^−1^ and N300: continuously N fed with five applications of 60 kg N ha^−1^ for a total of 300 kg ha^−1^.

The SEM micrographs of the straw stem section show different appearances depending on the wheat cultivar and crop N fertilization treatment (Figure 1). For both cultivars, but especially for Bologna, the number of cell layers in the stem is much lower in treatments with low and early N availability (treatments N0 and N60+0) than in the treatment that was constantly well N fed (N300), while an intermediate situation can be observed in the treatment with only late N availability. Apart from the different number of cell layers, the stem structure appears substantially similar in all treatments in terms of the number of vessels and the appearance of metaxylem, protoxylem and fibers. No differences in cellulose and lignin abundances in cell walls can be argued from the micrographs. Unfortunately, while the effect of the N fertilization rate on stem structure is quite well known [31], there is no literature on the combined effect of the N fertilization rate and application timing on stem structure and composition. It is undeniably well understood that the mechanical properties of a composite are strongly related to the inherent characteristic of both the matrix and the reinforcing agent; however, straw characteristics are variable and essentially related to different factors, such as plant variety, stem dimension, plant maturity and moisture content; additionally, they are also dissimilar at diverse heights of the stem. In our investigation, the straw samples collected from each cultivar were bulked and chopped, so the correlation of the main characteristics of the crop stalks, such as length–diameter ratio, with the mechanical performance of the produced short-fiber random composite material loses, as a matter of fact, significance.

### 3.2. Straw Composition

The analysis of the fibrous fractions of straw carried out with the Van Soest method highlighted some differences among N fertilization treatments (Table 2). In particular, treatments with low and early N availability (N0 and N60+0) had higher mean ADF and cellulose contents as compared to treatments with high and late N availability (N300 and N0+120). Differences in the other fractions were small and/or apparently not related to N availability (e.g., ADL, which was low in BL_N0 and BR_N300).

### 3.3. X-Ray Diffraction Analysis of Straw

**Figure 2:**
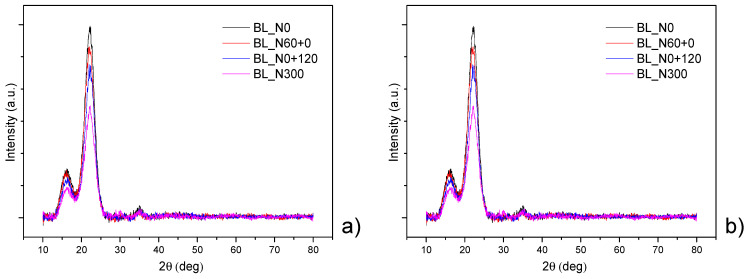
XRD patterns of straw for two wheat cultivars (Bologna, (**a**); Bora, (**b**)) grown with four nitrogen (N) fertilization treatments: N0: the unfertilized control, N60+0: N fed only very early with 60 kg N ha^−1^, N0+120: N fed only extremely late (i.e., at pollination) with 120 kg N ha^−1^ and N300: continuously N fed with five applications of 60 kg N ha^−1^ for a total of 300 kg ha^−1^.

X-Ray diffraction patterns (Figure 2) obtained from the straw of Bologna show remarkably similar trends with main peaks that decrease in intensity for N0+120 and N300. A similar trend, albeit less intense, is also noted for Bora straw. The XRD diffractograms show a strong main peak around 22° with a shoulder in the range of 14–17° and another weaker peak near 34°, which are characteristic diffraction patterns of crystalline cellulose [32,33]. A lower intensity of the main peak with late or high N availability indicates a decrease in crystallinity. In particular, the three main crystalline areas centered at 2θ = 14–17° relative to the (101) and (10^−^;1) planes; the overriding peak at 2θ = 22° with respect to the (002) plane and a peak area weak at 2θ = 34° attributable to the (040) plane are compatible with predominantly type I crystalline cellulose [32,34].

This parameter, together with the cellulose fraction, is crucial for the evaluation of mechanical properties for biocomposites, as the stiffness increases with crystalline cellulose content. The crystallinity index (*CI*) was determined using the amorphous subtraction method [35]. As reported by Park et al. [35] once the amorphous area (*I_a_*) and the crystalline area (*I_c_*) were defined, the CI was calculated as the ratio between the crystalline area and the total area (Equation (1)):

(1) $$Crystallinity\;Index\;\left(CI\right)=\frac{I_{crystalline}}{I_{amorphous}+I_{crystalline}}\times 100\%$$

where *I_crystalline_* (*I_c_*) corresponds to the crystalline peaks’ integrated areas and *I_amorphous_* (*I_a_*) is the amorphous background integrated area, after zeroing to the baseline [35,36]

**Figure 3:**
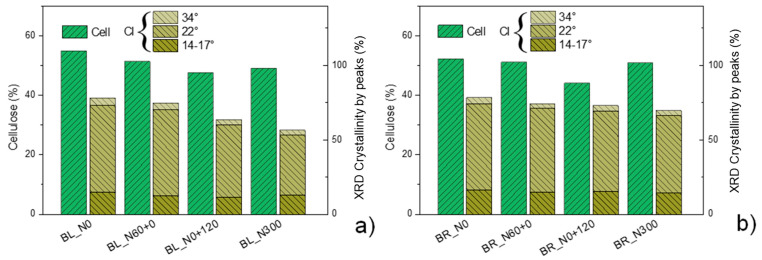
Cellulose content according to the Van Soest method and the crystallinity index (CI) calculated from XRD (as sum of the three main crystalline peaks) for the straw of the two cultivars Bologna (**a**) and Bora (**b**) grown with four nitrogen (N) fertilization schedules: N0: the unfertilized control, N60+0: N fed only very early with 60 kg N ha^−1^, N0+120: N fed only extremely late (i.e., at pollination) with 120 kg N ha^−1^ and N300: continuously N fed with five applications of 60 kg N ha^−1^ for a total of 300 kg ha^−1^.

In Figure 3, the cellulose content obtained with the Van Soest method for the straw of the two cultivars Bologna and Bora grown with different N fertilization treatments is shown in histograms alongside the respective crystalline index obtained via XRD, dividing the contributions for the three main crystalline peaks.

In the case of Bologna straw, late and high N fertilization caused a decrease in the cellulose content, and at the same time, the crystallinity index decreases. A similar trend, although less evident, was also noted for Bora [10].

The morphology of the refined flours obtained from the two wheat cultivars, Bologna and Bora, at different nitrogen fertilization schedules, as well as the morphology of the materials obtained from them via thermoplasticization, was studied in our previous work [8]. Results had shown that the most balanced mechanical performance for each of the two cultivars was obtained with flours of crops grown with constantly high N availability (N300). For this reason, the thermoplastic matrices obtained from the neat flours of the well-fertilized crops (BL-N300 and BR-N300) were used here to produce the biocomposites reinforced with the straws obtained from crops grown with different N fertilization treatments.

**Figure 4:**
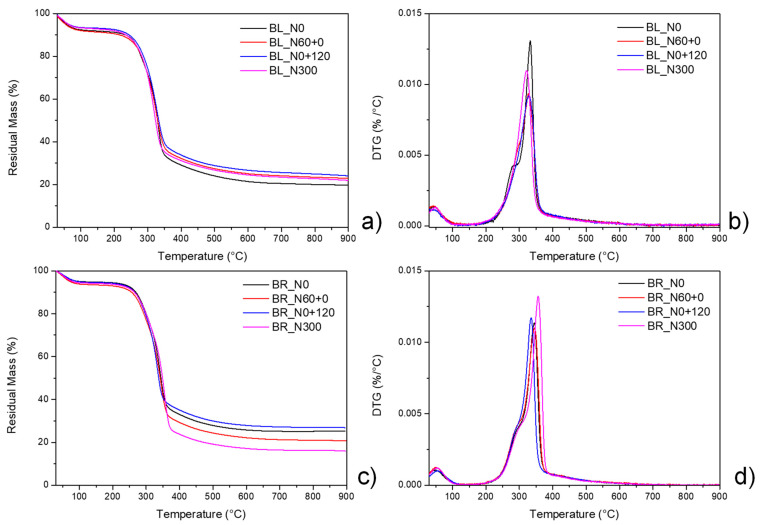
TG/DTG curves of straw for two cultivars (Bologna, (**a**,**b**); Bora, (**c**,**d**)) of bread wheat grown with four nitrogen (N) fertilization treatments: N0: the unfertilized control, N60+0: N fed only very early with 60 kg N ha^−1^, N0+120: N fed only extremely late (i.e., at pollination) with 120 kg N ha^−1^, N300: continuously N fed with five applications of 60 kg N ha^−1^ for a total of 300 kg ha^−1^.

The thermal degradation behavior of wheat straw was analyzed via thermogravimetric analysis (TGA). The mass loss (TG) curves for the Bologna (Figure 4a) and Bora straws (Figure 4c) are quite similar, regardless of the N fertilization treatments. Differential thermogravimetric (DTG) curves, which give the rate of weight loss for each temperature, show three main weight losses for both Bologna (Figure 4b) and Bora (Figure 4d) straws.

The water contained in the fibers appears with a maximum mass loss rate of around 50–60 °C. The second peak, which starts at 200 °C, attributable to the decomposition of hemicellulose and sugars, is partially overlapped by the decomposition peak of cellulose and, at higher temperatures, by that of the lignin component. The temperatures of the decomposition peaks for hemicellulose and cellulose can be considered to be around 260 °C and around 340 °C, respectively; sugar biomass decomposes at 250–350 °C, while lignin has a decomposition temperature range of 250–800 °C based on its different structures, although most thermal degradation occurs between 360 and 500 °C [37,38,39].

The variations in the decomposition curves of the straw of different treatments (cultivars and fertilization management) are mainly attributable to differences in humidity and in the ratio between straw constituents (hemicellulose, cellulose, polysaccharides, lignin, etc.) [37] Residual mass values at 900 °C in the range of 15–30% are characteristic of the residual ash of wheat straw; their variability depends on the composition of the fibrous quotas and the heterogeneity of the biological samples [40,41].

**Figure 5:**
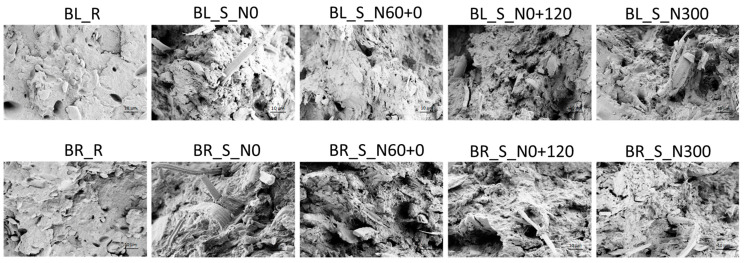
FESEM micrographs of thermoplastic bulk samples obtained from refined (R) wheat flours of the cultivars Bologna (BL) and Bora (BR) whose crops were continuously nitrogen (N) fed with five applications of 60 kg N ha^−1^ for a total of 300 kg N ha^−1^ (samples BL_R and BR_R) or from the same flours added with straw (S) (15% *w*:*w*) obtained from crops fertilized with four different N fertilization treatments: N0: the unfertilized control (samples BL_S_N0 and BR_S_N0), N60+0: N fed only very early with 60 kg N ha^−1^ (samples BL_S_N60+0 and BR_S_ N60+0), N0+120: N fed only extremely late (i.e., at pollination) with 120 kg N ha^−1^ (samples BL_S_N0+120 and BR_S_N0+120) and N300: continuously N fed with five applications of 60 kg N ha^−1^ for a total of 300 kg ha^−1^ (samples BL_S_N300 and BR_S_N300).

In Figure 5, the fractured surfaces of thermoplastic bulk samples derived from refined flour of both Bologna and Bora N300 appear relatively smooth, with the starch granules well interfaced, which is representative of good compactness. The homogeneous surfaces free of cracks and with only some agglomerated particles indicate strong interfacial adhesion between proteins and the thermoplastic matrix, suggesting good mechanical performance for both matrices BL_R and BR_R. All the micrographs of the biocomposites show good adhesion between straw-reinforcing fibers and plasticized flour matrices, indicating good compatibility between these biomaterials.

**Figure 6:**
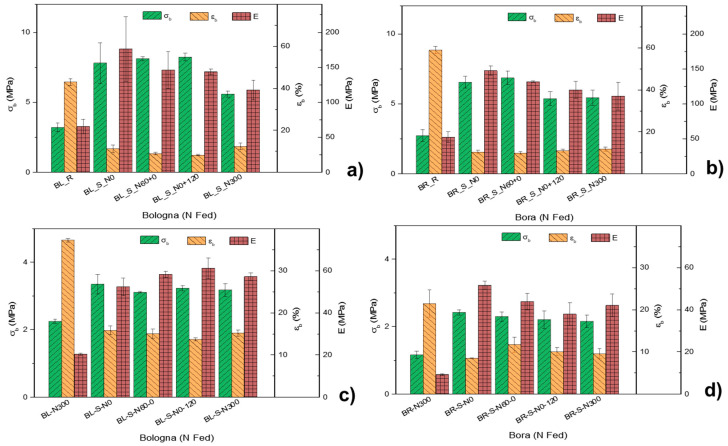
Tensile properties (tensile strength at break, σ_b_; elongation at break, ε_b_; and Young’s modulus, E) of thermoplastic bulk samples obtained from refined (R) wheat flours of the cultivars Bologna (BL, left at (**a**))11RH and (**c**)) 53RH) and Bora (BR, right at (**b**)) 11RH and (**d**)) 53RH) whose crops were continuously nitrogen (N) fed with five applications of 60 kg N ha^−1^ for a total of 300 kg N ha^−1^ (samples BL_R and BR_R) or from the same flours added with straw (S) (15% *w*:*w*) obtained from crops fertilized with four different N fertilization treatments: N0: the unfertilized control (samples BL_S_N0 and BR_S_N0), N60+0: N fed only very early with 60 kg N ha^−1^ (samples BL_S_N60+0 and BR_S_ N60+0), N0+120: N fed only extremely late (i.e., at pollination) with 120 kg N ha^−1^ (samples BL_S_N0+120 and BR_S_N0+120) and N300: continuously N fed with five applications of 60 kg N ha^−1^ for a total of 300 kg ha^−1^ (samples BL_S_N300 and BR_S_N300).

In the case of the absence of chemical pretreatments of the fibrous component, compatibility only relies on the presence of hydrogen bonds that could cause the plant fiber to bind with starch tightly to form a homogeneous phase; here, we have assumed that different N treatments do not give any surface variation to the straw, and accordingly, any variation in the final performance of the composites can be due to the overall composition (in terms of cellulose, hemicellulose and lignin) and stem morphology. Indeed, for both varieties, the mechanical properties of the composites can be traced back to the different morphologies and compositions of the straws with different N fertilization treatments. As highlighted by the SEM micrographs of the straws (Figure 1, N0 and N300), the N300 straws appear much thicker and more massive than the N0 ones, so with the same weight of added reinforcing fibers (15% *w*:*w*) in the biocomposite, the N0 straws were more numerous than the N300 ones. The higher cellulose content with a higher crystalline index value makes the unfertilized straws more resistant. Furthermore, the lower number of cell layers of N0, as compared to N300 (Figure 1), corresponds to a thinner morphological structure, implying a greater flexibility that reduces fragmentation during mixing with the matrix. On the other hand, the straw fibers of the late N-fertilized crop (N0+120) appear more fragmented in the composites. This is due to their less resistant composition (with less crystalline cellulose) and more massive structure, and they tend to fragment more due to the shear stresses generated during the melt mixing process used to produce the biocomposites. The mechanical behavior of bioplastics is generally affected by ambient humidity, because thermoplastic starches (and flours) absorb moisture when exposed to humidity. Moreover, the incorporation of abundant but intrinsically hydrophilic lignocellulosic materials can modify via reduction both strength and toughness in these matrices, because ambient humidity can affect the properties of the fiber as well. Accordingly, the performance of biocomposites can be significantly affected by ambient humidity. For this reason, two different conditionings of the thermoplasticized composites were carried out before the tensile test for 48 h at 23 °C at two different air relative humidity levels (11% and 53% RH) to simulate the natural operating conditions of the composites in dry and humid conditions; the graphs, representing the tensile strength (σ_b_), the strain at break (ε_b_) and the Young’s modulus (E), obtained with the tensile tests on the samples conditioned at (a) 11% and (b) 53% relative humidity (RH) are shown in Figure 6.

Figure 6a and Table 3b show the results of the tensile tests for thermoplastic composites conditioned for 48 h at 11% RH. For both cultivars, the mechanical performance of plasticized biocomposites was improved when compared to plasticized refined flours. In general, the tensile strength (σ_b_) and Young’s modulus (E) were more than doubled, while ductility (ε_b_) decreased to less than a quarter. Among the composites, those obtained with the straw of crops grown under early and low N availability (N60+0 and N0) showed greater σ_b_ and E values than those obtained with the straw of crops grown under late and high N availability (N0+120 and N300). In general, the σ_b_ and E values obtained using the straw of N300 was the lowest among the composites. On the contrary, the ε_b_ value appeared not to follow a trend somehow related to the amount and timing of N fertilization. In particular, the tensile strength and the elastic modulus in Bologna-based composites were maximum for BL_S_N0 (σ_b_ = 8.41 ± 0.47 MPa; +163%) (E = 193 ± 35 MPa; +197%) compared to the BL_R matrix (σ_b_ = 3.20 ± 0.30 MPa; E = 65 ± 10 MPa). The ductility of the composites was significantly reduced, recording values between ε_b_ = 12.8% for BL_S_N300 and ε_b_ = 9.0% for BL_S_N60+0 compared to ε_b_ = 42.9% for the BL_R matrix. Similarly, in the Bora-based composites conditioned at 11% RH, the tensile strength and elastic modulus were maximum for the BR_S_N0 sample (σ_b_ = 6.64 ± 0.43 MPa; +143%; E = 147 ± 7 MPa; +183%) compared to the BR_R matrix (σ_b_ = 2.73 ± 0.42 MPa; E = 52 ± 8 MPa), while the ductility ranged between ε_b_ = 12.7% (BR_S_N300) and ε_b_ = 10.1% (BR_S_N60+0) compared to that of the BR_ R_N300 sample (ε_b_ = 58.8%). For both cultivars, the reinforcing effect of fibers was less effective with the straw of the late fertilized crop (N0+120).

The mechanical characterization of composite samples conditioned for 48 h at 53% RH shows a general reduction in tensile strength and elastic modulus compared to samples conditioned at 11% RH (Figure 6b and Table 3b), and this stands for both cultivars. This result is attributable to the hydrophilicity of these biocomposites, with water acting as a plasticizing agent [8]. However, even in thermoplastics conditioned at 53% RH, the composites show a significant improvement in performance as compared to the refined matrices, and differences among treatments substantially follow those observed for composites obtained at 11% RH. At 53% RH, the greatest increases in σ_b_ and E for Bologna composites, as compared to the thermoplastic obtained from the refined flour (BL_R), were recorded for BL_S_N300 (σ_b_ = 2.24 ± 0.06 MPa, +57%; E = 20.3 ± 0.4 MPa, +214%), while ε_b_ was more than halved for all composites (Δε_b_ = from −63% for BL_S_N0+120 to −58% for BL_S_N300, as compared to the value recorded for BL_R (ε_b_ 37.2 ± 0.4%). Similarly, in Bora, as compared to thermoplastics from the refined flour (BR_R: σ_b_ = 1.16 ± 0.11 MPa; E = 9.2 ± 0.4 MPa; ε_b_ =21.4 ± 3.2%), the greatest variations were recorded for BR_S_N0 (σ_b_ = +108%; E = +459%; ε_b_ = −58%). It is evident that, for both varieties at both air relative humidity levels, the best results in tensile strength and elastic modulus were obtained with straw from the crop grown under the lowest N availability (N0) condition.

**Figure 7:**
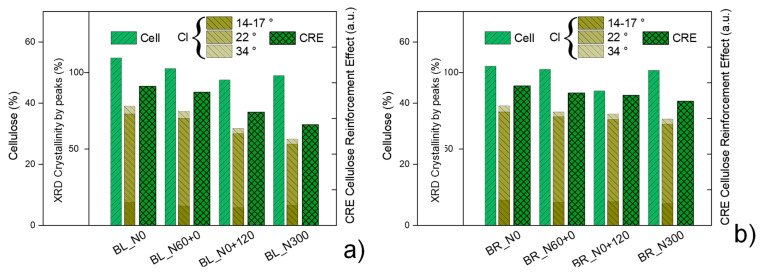
Cellulose content (Cell) and crystallinity Index (CI) used to obtain the cellulose reinforcement effect (CRE) of wheat straw of the two cultivars Bologna (**a**) and Bora (**b**) grown with four nitrogen (N) fertilization schedules: N0: the unfertilized control, N60+0: N fed only very early with 60 kg N ha^−1^, N0+120: N fed only extremely late (i.e., at pollination) with 120 kg N ha^−1^ and N300: continuously N fed with five applications of 60 kg N ha^−1^ for a total of 300 kg ha^−1^.

The results obtained with the mechanical characterization of biocomposites are consistent with the morphological analysis of their fracture surfaces obtained with FESEM micrographs (Figure 5). The different strengthening effect of straw harvested from different crop fertilization treatments can be explained by looking at the morphology and composition of each straw. The different N fertilization schedules induced straws with different compositions, specifically cellulose content and crystallinity (Figure 1 and Figure 3; Table 2). The straw harvested from crop grown under early and low N availability (N0 and N60+0) featured stems with a more resistant fibrous composition and a thinner and more flexible structure (Figure 1). These characteristics, combined with the greater number of fibers at the same weight, make the composites better reinforced and performing. On the other hand, late and high N fertilization (N0+120, N300) produced straw stems with a higher number of cell layers and larger vessels. This bulky morphology, combined with a lower crystalline cellulose content, induces the fragmentation of the reinforcing fibers and consequently less performing biocomposites [42,43]. It is worth noticing that the cellulose content or crystalline cellulose fraction, each considered separately, would not justify properly the obtained mechanical results. For example, BL_S_N300 showed significantly lower tensile strength than BL_S_N0+120, despite having a higher cellulose content, while the differences in elastic modulus between BR_S_N60+0 and BR_S_N300, which have similar cellulose contents, would not be justified. It can be assumed that, in order to exert a good reinforcing effect, the straw must have a high cellulose content, and this cellulose must have a high crystalline fraction. To simultaneously consider the effect of the cellulose content and its crystalline fraction, we tentatively introduced a fictitious coefficient given by the product between the cellulose content and its crystalline fraction, defining it as the cellulose reinforcement effect (CRE). If we consider the reinforcing effect of the straws as a function of this coefficient, we note a full correspondence between CI values in Figure 7 and the trend of the strength and Young modulus in Figure 6.

## 4. Conclusions

The results demonstrate that the tensile properties of biocomposites based on thermoplastic wheat flours reinforced with wheat straw depend on straw characteristics as they are affected by crop nitrogen availability. For both cultivars, the reinforcing effect of the straw was maximum when the straw came from crops grown with low and early N availability and minimum when the straw came from crops grown with high and late N availability. This improvement can be attributed mainly to the different straw morphologies, whereby, with poor nitrogen availability, stem vessels are more compact, with higher proportions of cellulose and higher crystalline fractions, and therefore possess higher strength values for the composite. The performance of the biocomposites was also significantly affected by the ambient relative humidity at which samples were stabilized just before tensile tests. In samples stabilized for 48 h at 53% relative humidity, as compared to 11% RH, the tensile strength of the same samples was more than halved, and the elastic modulus was reduced to about one-third. However, the reinforcing effect of straw with respect to refined matrices was evident also at high humidity (53% RH), and the differences among composites in tensile properties due to different straw sources were maintained. (The lower and earlier the N availability of the crop, the greater the tensile strength and Young’s modulus).

This evidence further confirms that plant-based materials engineering needs to carefully take into consideration the variability of source material characteristics, which depend not only on the plant species and cultivated variety but also on crop growing conditions, with the nitrogen fertilization rate and schedule as major drivers.

## Figures and Tables

**Table 1:** Formulations of biocomposites based on refined (R) wheat flours of the cultivars Bologna (BL) and Bora (BR) whose crops were continuously nitrogen (N) fed with five applications of 60 kg N ha^−1^ for a total of 300 kg N ha^−1^ or based on the same flours added with straw (S) (15% *w:w*) obtained from crops fertilized with four different N fertilization treatments: N0: the unfertilized control, N60+0: N fed only very early with 60 kg N ha^−1^, N0+120: N fed only extremely late (i.e., at pollination) with 120 kg N ha^−1^ and N300: continuously N fed with five applications of 60 kg N ha^−1^ for a total of 300 kg ha^−1^.

Dough Label	Description
BL_R	Bologna refined flour of N300 (without straw)
BL_S_N0	Bologna flour of N300 added with straw of N0
BL_S_N60+0	Bologna flour of N300 added with straw of N60+0
BL_S_N0+120	Bologna flour of N300 added with straw of N0+120
BL_S_N300	Bologna flour of N300 added with straw of N300
BR_R	Bora refined flour of N300 (without straw)
BR_S_N0	Bora flour of N300 added with straw of N0
BR_S_N60+0	Bora flour of N300 added with straw of N60+0
BR_S_N0+120	Bora flour of N300 added with straw of N0+120
BR_S_N300	Bora flour of N300 added with straw of N300

**Table 2:** Compositions (g 100 g^−1^) of wheat straw of the two cultivars Bologna and Bora grown with four nitrogen (N) fertilization treatments: N0: the unfertilized control, N60+0: N fed only very early with 60 kg N ha^−1^, N0+120: N fed only extremely late (i.e., at pollination) with 120 kg N ha^−1^ and N300: continuously N fed with five applications of 60 kg N ha^−1^ for a total of 300 kg ha^−1^. DM, dry matter; NDF, neutral detergent fiber; ADF, acid detergent fiber; Cell, cellulose; ADL, acid detergent lignin; CP, crude protein.

Cultivar	N Fert	DM	NDF	ADF	Cell	ADL	CP
BL	N0	94.12	75.63	59.00	54.70	4.30	3.72
BL	N60+0	94.42	73.35	58.74	51.23	7.51	3.52
BL	N0+120	94.63	73.50	55.76	47.50	8.26	3.72
BL	N300	94.33	74.44	56.51	48.96	7.55	4.84
BR	N0	94.77	73.27	59.11	52.02	7.09	2.29
BR	N60+0	95.42	72.91	57.76	50.94	6.82	2.12
BR	N0+120	94.98	70.24	51.85	43.91	7.94	4.81
BR	N300	94.72	75.18	55.62	50.67	4.95	4.84

**Table 3:** Mechanical properties (tensile strength, σ_b_; elongation at break, ε_b_; and Young’s modulus, E) of the tensile tests carried out on thermoplastic bulk samples obtained from refined (R) wheat flours of the cultivars Bologna (BL, left) and Bora (BR, right) whose crops were continuously nitrogen (N) fed with five applications of 60 kg N ha^−1^ for a total of 300 kg N ha^−1^ (samples BL_R and BR_R) or from the same flours added with straw (S) (15% *w:w*) obtained from crops fertilized with four different N fertilization treatments: N0: the unfertilized control (samples BL_S_N0 and BR_S_N0), N60+0: N fed only very early with 60 kg N ha^−1^ (samples BL_S_N60+0 and BR_S_ N60+0), N0+120: N fed only extremely late (i.e., at pollination) with 120 kg N ha^−1^ (samples BL_S_N0+120 and BR_S_N0+120) and N300: continuously N fed with five applications of 60 kg N ha^−1^ for a total of 300 kg ha^−1^ (samples BL_S_N300 and BR_S_N300). Bioplastic composites, before the tensile test, were stabilized at two different air relative humidity (RH) levels for 48 h at 23 °C: (**a**) 11% RH and (**b**) 53% RH. The results obtained are reported in Table 3a for 11% RH and in Table 3b for 53% RH.

**(a)**			
**Sample (11RH)**	**σ_b_ (MPa)**	**ε_b_ (%)**	**E (MPa)**
BL_R_N300	3.20 ± 0.30 ^a^	42.9 ± 1.6 ^a^	65 ± 10 ^a^
BL_S_N0	8.41 ± 0.47 ^c^	12.0 ± 1.8 ^bc^	193 ± 35 ^c^
BL_S_N60+0	8.09 ± 0.16 ^c^	9.0 ± 0.5 ^c^	177 ± 18 ^c^
BL_S_N0+120	7.85 ± 0.16 ^c^	9.3 ± 1.8 ^bc^	168 ± 22 ^c^
BL_S_N300	5.63 ± 0.19 ^b^	12.8 ± 1.6 ^b^	120 ± 13 ^b^
BR_R_N300	2.73 ± 0.42 ^a^	58.8 ± 1.8 ^a^	52 ± 8 ^a^
BR_S_N0	6.64 ± 0.43 ^b^	10.9 ± 0.6 ^b^	147 ± 7 ^c^
BR_S_N60+0	6.52 ± 0.47 ^b^	10.1 ± 0.7 ^b^	131 ± 1 ^bc^
BR_S_N0+120	5.45 ± 0.56 ^b^	11.5 ± 0.7 ^b^	120 ± 12 ^bc^
BR_S_N300	5.61 ± 0.69 ^b^	12.7 ± 1.2 ^b^	111 ± 20 ^b^
**(b)**			
**Sample (53RH)**	**σ_b_ (MPa)**	**ε_b_ (%)**	**E (MPa)**
BL_R	2.24 ± 0.06 ^a^	37.2 ± 0.4 ^a^	20.3 ± 0.4 ^a^
BL_S_N0	3.51 ± 0.11 ^c^	15.2 ± 0.4 ^b^	63.8 ± 2.2 ^c^
BL_S_N60+0	3.23 ± 0.17 ^bc^	15.6 ± 0.3 ^b^	60.9 ± 1.1 ^c^
BL_S_N0+120	3.12 ± 0.06 ^b^	13.6 ± 0.4 ^c^	58.4 ± 1.2 ^c^
BL_S_N300	3.04 ± 0.12 ^b^	15.8 ± 0.4 ^b^	51.3 ± 1.1 ^b^
BR_R	1.16 ± 0.11 ^a^	21.4 ± 3.2 ^a^	9.2 ± 0.4 ^a^
BR_S_N0	2.41 ± 0.08 ^b^	8.9 ± 0.5 ^b^	51.4 ± 1.9 ^c^
BR_S_N60+0	2.36 ± 0.12 ^b^	10.7 ± 0.8 ^b^	45.7 ± 2.5 ^bc^
BR_S_N0+120	2.20 ± 0.26 ^b^	10.0 ± 1.0 ^b^	37.8 ± 5.4 ^b^
BR_S_N300	2.08 ± 0.10 ^b^	9.9 ± 1.4 ^b^	35.7 ± 2.2 ^b^

(a–c) Different superscripts for each cultivar within the same column indicate significant differences (*p* < 0.05) among the five formulations.

## Data Availability

The original contributions presented in this study are included in the article. Further inquiries can be directed to the corresponding author.
